# The Influence of the Nature of the Polymer Incorporating the Same A_3_B Multifunctional Porphyrin on the Optical or Electrical Capacity to Recognize Procaine

**DOI:** 10.3390/ijms242417265

**Published:** 2023-12-08

**Authors:** Anca Lascu, Dana Vlascici, Mihaela Birdeanu, Camelia Epuran, Ion Fratilescu, Eugenia Fagadar-Cosma

**Affiliations:** 1 Institute of Chemistry “Coriolan Dragulescu”, Mihai Viteazu Ave. 24, 300223 Timisoara, Romania; alascu@acad-icht.tm.edu.ro (A.L.); ecamelia@acad-icht.tm.edu.ro (C.E.); ionfratilescu@acad-icht.tm.edu.ro (I.F.); 2 Faculty of Chemistry, Biology, Geography, West University of Timisoara, 4 Vasile Parvan Ave., 300223 Timisoara, Romania; dana.vlascici@e-uvt.ro; 3 National Institute for Research and Development in Electrochemistry and Condensed Matter, Plautius Andronescu Street 1, 300224 Timisoara, Romania; mihaelabirdeanu@gmail.com

**Keywords:** carboxy-substituted tolylporphyrin, k-carrageenan, polyvinylchloride membrane, procaine detection, optical detection, ion-selective membrane electrode

## Abstract

The multifunctionality of an A_3_B mixed-substituted porphyrin, namely 5-(4-carboxyphenyl)-10,15,20-tris(4-methylphenyl)porphyrin (5-COOH-3MPP), was proven due to its capacity to detect procaine by different methods, depending on the polymer matrix in which it is incorporated. The hybrid nanomaterial containing k-carrageenan and AuNPs (5-COOH-3MPP-k-carrageenan-AuNPs) was able to optically detect procaine in the concentration range from 5.76 × 10^−6^ M to 2.75 × 10^−7^ M, with a limit of detection (LOD) of 1.33 × 10^−7^ M. This method for the detection of procaine gave complementary results to the potentiometric one, which uses 5-COOH-3MPP as an electroactive material incorporated in a polyvinylchloride (PVC) membrane plasticized with o-NPOE. The detected concentration range by this ion-selective membrane electrode is wider (enlarged in the field of higher concentrations from 10^−2^ to 10^−6^ M), linearly dependent with a 53.88 mV/decade slope, possesses a detection limit of 7 × 10^−7^ M, a response time of 60 s, and has a certified stability for a working period of six weeks.

## 1. Introduction

Procaine is a well-known local anesthetic used since its synthesis in 1905 for oral medicine [1] and for veterinary use [2]. In addition, procaine has been extensively studied, and it was discovered that this molecule displayed beneficial effects on the functions of cells as an antioxidant, cytoprotective, and anti-inflammatory agent [3]. The radioprotective effect of procaine on human lymphocytes after ionizing radiation has been recently documented [4]. All these results justify the use of procaine-based drugs for the prophylaxis and treatment of metabolic and degenerative processes that occur in elderly people. Knowing that the administered concentrations of procaine in local anesthesia are 4.23 × 10^−4^ M and the highest safety limit is 4.23 × 10^−2^ M in general anesthesia [5], the two proposed methods presented in this work are both highly suited for human medical monitoring. Thus, the detection and precise quantitation of procaine are of ongoing research interest. Some of the recently reported detection methods, the detected concentration domain, and the limits of detection are summarized in Table 1.

Porphyrins alone or associated with other chromophores have been intensively used in sensor formulations for extended biomedical analysis. A symmetrical carboxyphenyl substituted porphyrin, namely, 5,10,15,20-tetrakis-(4-carboxyphenyl)porphyrin, has been previously reported in MOF preparations and acts as a dual-mode electroluminescent sensor for the detection of the anti-inflammatory drug S-naproxen [15]. A conjugate system made from zinc-porphyrin and cinnamic acid was used as a sensitive compound in a fluorescence sensor for theophylline quantification [16]. Trans-diaxial heterodimers constituted from porphyrins and highly emissive fluorescein are reported as alternative variants to monitor pH changes from fluorescence [17].

New genosensors based on electrochemical markers made from Co(II/III)-metalloporphyrin-modified DNA attached to AuNPs/AgNPs were able to specifically detect DNA sequences in the femtomolar range [18]. Another DNA detection method using AuNPs employs fluorescence microscopy [19], which can discriminate between the processes of DNA immobilization, hybridization, or degradation.

A small number of organic polymers might be used to create synergistic effects in combination with porphyrins and prevent porphyrin aggregation [20] as well as inactivation, such as polyvinylchloride, chitosan [21,22], cellulose [23], β-(1,3-1,6)-D-glucan, λ-carrageenan, tamarind gum, and pullulan [24].

An overview regarding the use of polymer-porphyrin composite materials in sensor design is presented in Table S1. The listed materials are able to detect biologically active compounds through various methods.

**Figure 1:**
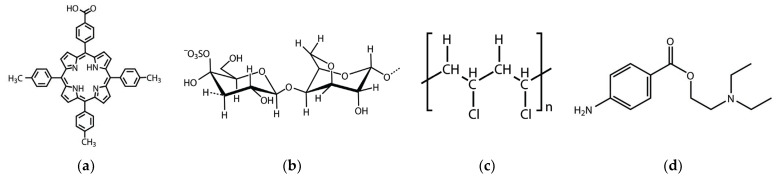
Chemical structures of: (**a**) 5-(4-carboxyphenyl)-10,15,20-tris(4-methylphenyl)porphyrin (5-COOH-3MPP), (**b**) k-carrageenan, (**c**) polyvinylchloride (PVC), and (**d**) procaine.

In the last few years, our group has focused on finding diverse applications by using the same porphyrin structure. In this respect, a mixed substituted A_3_B porphyrin, namely, 5-(4-carboxyphenyl)-10,15,20-tris(4-methylphenyl)porphyrin (5-COOH-3MPP) presented in Figure 1a, was successfully designed and applied for CO_2_ capture [25] as well as a corrosion inhibitor for steel in acid media [26]. 

The aim of this work is to emphasize the response versatility of this A_3_B porphyrin structure, which contains a carboxylic functional group, in facilitating procaine recognition/detection by means of physical-chemical interactions with NH_2_ groups present in the molecule of the analyte. The same porphyrin structure was sensitive in both optical and potentiometric detection by changing the polymer in which it was incorporated. Figure 1b,c depict the structures of the used polymers. Figure 1d presents the structure of the investigated analyte, namely procaine.

A_3_B porphyrin, when used as a sensing material, demonstrated to be multifunctional, proving its ability to detect procaine potentiometrically when incorporated in a polyvinylchloride (PVC) membrane as well as optically when involved in a hybrid nanomaterial containing k-carrageenan and AuNPs that amplifies its optical properties. The intended purpose of the reported research is to propose cost-effective and simple methods to detect procaine.

**Figure 2:**
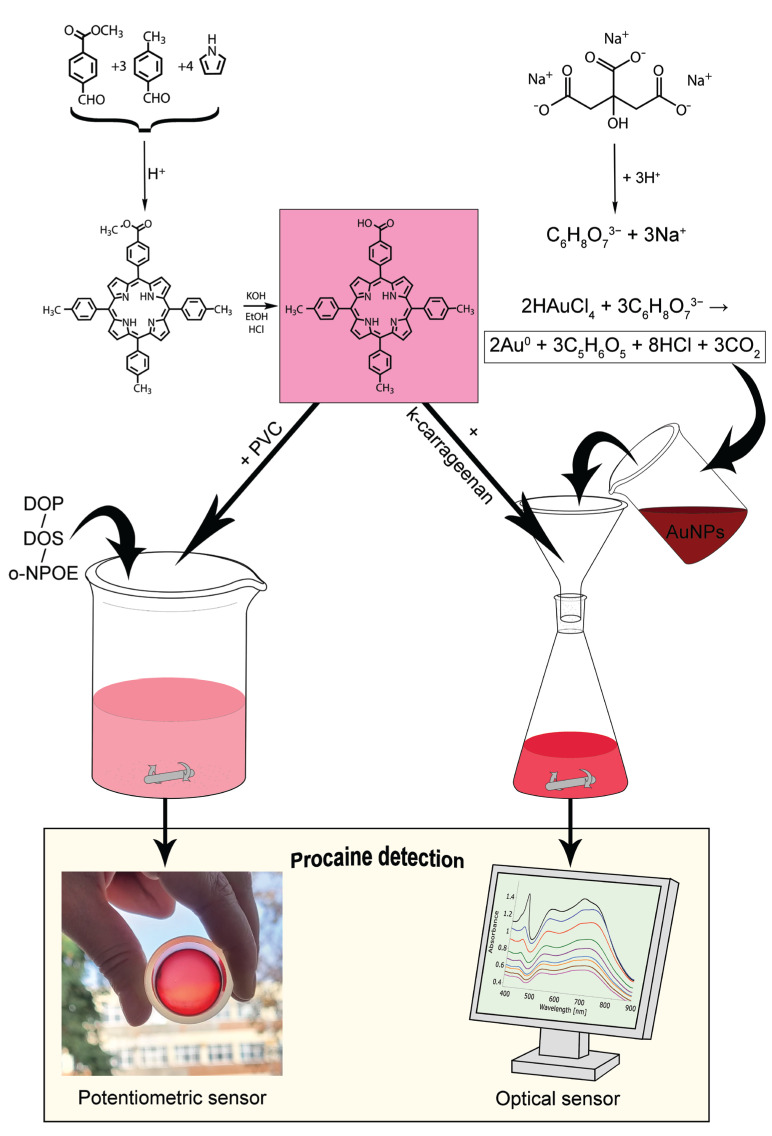
A diagram detailing the synthesis of porphyrin, formulation of 5-COOH-3MPP-k-carrageenan-AuNPs nanomaterial, fabrication of procaine ion-selective membrane, and final applications in potentiometric and optical sensing.

A diagram portraying our main targets: the synthesis of porphyrin, the obtainment of 5-COOH-3MPP-k-carrageenan-AuNPs nanomaterials, and the fabrication of procaine ion-selective membranes, together with their further sensing applications, is drawn in Figure 2.

## 2. Results and Discussion

### 2.1. Optical Detection of Procaine with 5-COOH-3MPP-k-Caragenan-AuNPs Nanomaterial

A volume of 5 mL of the complex nanomaterial 5-COOH-3MPP-k-caragenan-AuNPs in the DMF/water mixture = 1/9 was acidulated to pH = 2 with HCl (c = 37%). In decade-prepared solutions of procaine in water (v = 0.3 mL), starting from c = 1 × 10^−5^ M, were added to the nanomaterial solution, stirred for 1 min, and then the UV-vis spectra were recorded.

**Figure 3:**
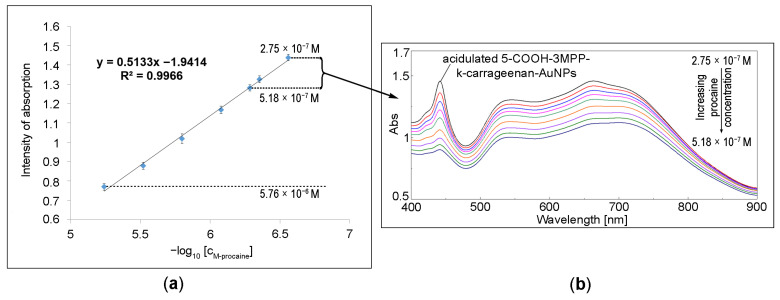
(**a**) The linear dependence between the absorption intensity read at 650 nm and the procaine concentration; (**b**) The detail of UV-vis spectra during the optical detection of procaine by 5-COOH-3MPP-k-caragenan-AuNPs composite material in the field of trace concentrations.

The full concentration of procaine that was linearly detected by the optical method ranged from 5.76 × 10^−6^ M to 2.75 × 10^−7^ M (Figure 3a,b), with a limit of detection (LOD) of 1.33 × 10^−7^ M (calculated using Equation (1)) and a sensitivity of 9.01 µmol procaine solution/A.U intensity (calculated using Equation (2)).

Equation (1), used to calculate the value of the LOD, is as follows:

LOD = 3σ/K(1)

where σ is the standard deviation of the blank measurement and K is the slope between the absorbance and the analyte concentration [27].

Equation (2) was used to obtain the sensitivity value that represents the smallest amount of substance in a sample that can be accurately measured by an assay [28].

(2) $$SENSITIVITY=\frac{\Delta C}{\Delta I}$$

where ∆*C* represents the difference in the procaine concentration in two successive samples expressed in micromolar units, and ∆*I* is the difference between intensities of absorption in the same two samples.

#### The Effects of Interferent Species in the Optical Detection of Procaine

**Figure 4:**
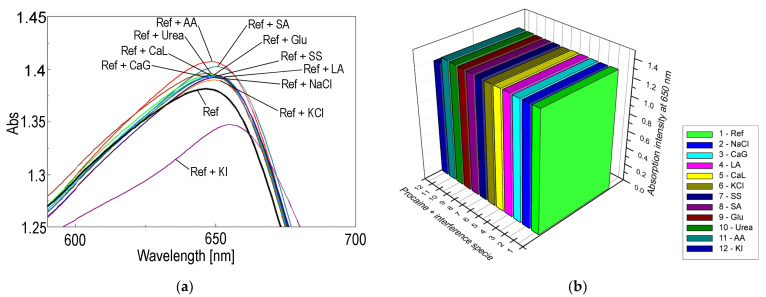
(**a**) The overlapped UV-vis spectra showing the insignificant effects of interferent species (excepting the moderate effect of KI) and confirming the selectivity of procaine detection. (**b**) 3D plot representing the absorption intensity differences induced by interferent compounds at λ = 650 nm.

**Figure 5:**
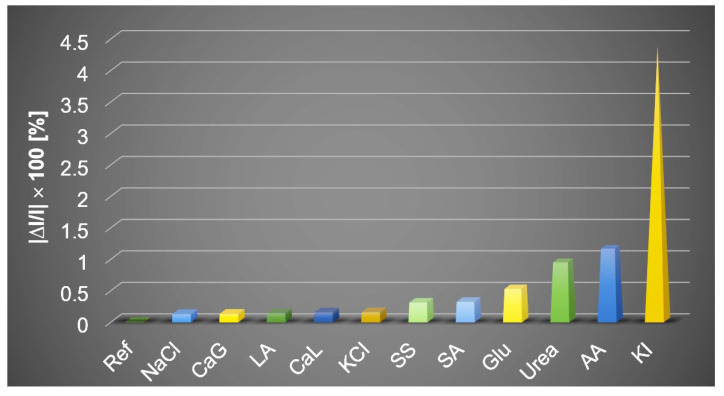
A representation of the average percentage error induced by several interference compounds (in concentrations exceeding 10 times the procaine concentration).

In order to confirm the selectivity of detection for procaine, tests for potential interferent species were performed, selecting NaCl, KI, Ascorbic acid, Lactic acid, Ca lactate, Ca gluconate, Na acetate, glucose, urea, Na salicilate, that are the most probably present in the medical samples. The overlapped UV-vis spectra are depicted in Figure 4a. The control sample (Ref) consists of acidulated 5-COOH-3MPP-k-carrageenan-AuNPs in a DMF-water system in which the concentration of procaine is c = 1 × 10^−6^ M. The interferent solutions contain the same concentration of procaine as in Ref and a ten-fold higher concentration of each interferent species. It can be concluded that the only significant interferent species is produced by iodide anion (<5% deviation), which is represented in the 3D plot in Figure 4b. The average percentage errors given by each of the interfering compounds are drawn in Figure 5.

### 2.2. FT-IR Analysis for Presuming the Mechanism of Detection

**Figure 6:**
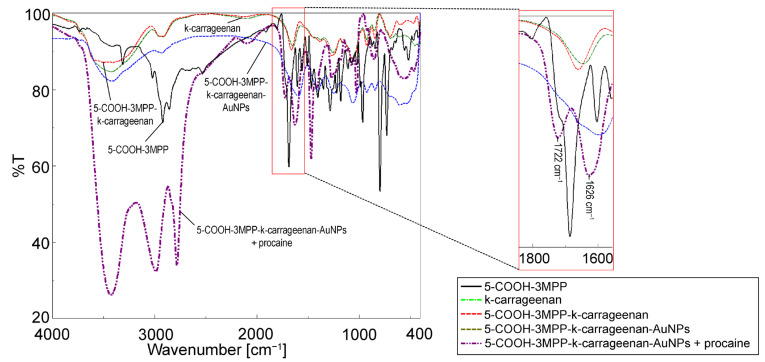
The overlapped FT-IR spectra for: 5-COOH-3MPP, k-carrageenan, 5-COOH-3MPP-k-carrageenan, 5-COOH-3MPP-k-carrageenan-AuNPs, and 5-COOH-3MPP-k-carrageenan-AuNPs after interaction with procaine.

The FT-IR spectra for procaine and k-carrageenan are well-known [29,30], and the spectrum for 5-COOH-3MPP was completely presented and characterized in our previous paper [25]. The FT-IR spectra of these enumerated compounds are comparatively presented in Figure 6 with the spectrum of the hybrid material 5-COOH-3MPP-k-carrageenan-AuNPs after being exposed to procaine in order to reveal the structural modifications during the detection process.

The composite nanomaterial 5-COOH-3MPP-k-carrageenan-AuNPs, after exposure to procaine, reveals the characteristic peaks for each component and the new ones formed due to interaction with procaine in the FT-IR spectrum. The peak at 462 cm^−1^ can be presumed to be indicative of the bond between gold and the carbon atom (νC-Au) [31]; the peak located at 856 cm^−1^ reveals the νC-O-S bond from k-carrageenan; the characteristic peak at 1024 cm^−1^ indicates a pyran ring in polysaccharides [32,33]; and the signal at 1072 cm^−1^ is indicative of the νC-O-C glycosidic bond from k-carrageenan [32]. The porphyrin also gives specific signals at 1181 cm^−1^ representing the (δC-Hpyrrole) bond. Another peak at 1267 cm^−1^ is assigned to the νC-O bond in porphyrin but overlaps with the νO=S=O bond in k-carageenan. Other peaks that confirm the presence of porphyrin are 1467 cm^−1^ (νC=N). The most intense signal around 1660 cm^−1^ represents the (νC=C) vibration in porphyrin.

The presence of k-carrageenan is confirmed by the peak at 2780 cm^−1^, indicative of the νC-H bond in a polymer [34,35]. The abundant C–H bonds present large stretching vibrations at 2988 cm^−1^ [36], and the many OH groups are confirmed by the widened peak located at 3426 cm^−1^, which is typical for intrabackbone H-bonds.

#### Presumed Mechanism of Optical Detection

Two new and distinctive peaks were clearly formed after the interaction with procaine at 1626 cm^−1^ and 1722 cm^−1^, as presented in magnified detail in Figure 6. The first peak is assigned to porphyrin diminished νC=O bonding strength after linking to procaine, and the second one represents the signal for negatively charged nitrogen from procaine during electrostatic interaction with positively charged hydrogen from the COOH group grafted on the porphyrin (a chemical relationship between the donating amino group and the accepting carboxyl group) [37].

### 2.3. AFM Analysis to Evidence Morphological Changes after 5-COOH-3MPP-k-Carrageenan-AuNPs Material Interaction with Procaine

#### 2.3.1. AFM of 5-COOH-3MPP-k-Carrageenan-AuNPs Nanomaterial

**Figure 7:**
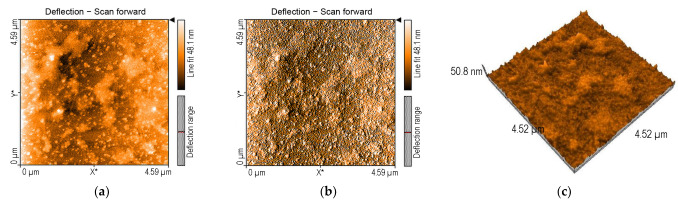
(**a**) A 2D image for 5-COOH-3MPP-k-carrageenan-AuNPs material. (**b**) AFM image in shadows (**c**) A 3D image of the same area and magnitude.

Figure 7 shows the surface morphology of the 5-COOH-3MPP-k-carrageenan-AuNPs material before exposure to procaine.

The surface of the silica plate is unevenly covered with large zig-zag aggregates, surrounded by spherical gold nanoparticles. The high value of the rugosity Sa (5464 nm) of the complex mixture can explain the facilitated access of the procaine molecules to the recognition sites of the 5-COOH-3MPP-k-carrageenan-AuNPs nanomaterial.

#### 2.3.2. AFM of Composite Nanomaterial 5-COOH-3MPP-k-Carrageenan-AuNPs after Exposure to Procaine

**Figure 8:**
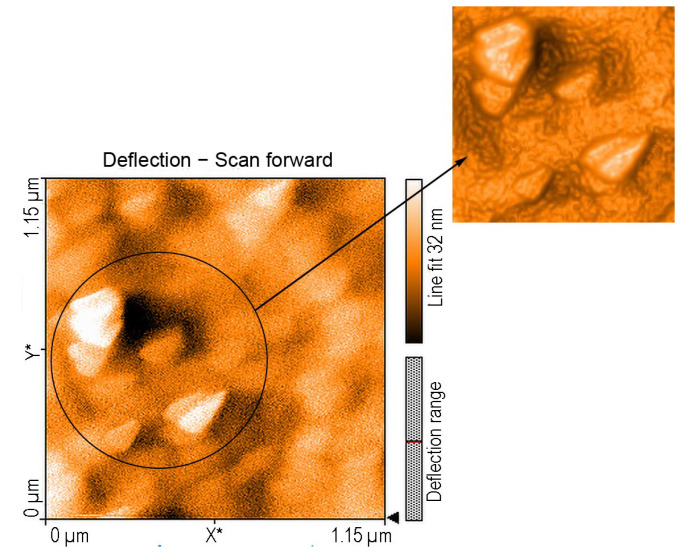
A 2D image of 5-COOH-3MPP-k-carrageenan-AuNPs material after exposure to procaine with 3D detail of the same material confirming both H- and J-type aggregation and the non-uniform surface with large voids.

The atomic force microscopy images for 5-COOH-3MPP-k-carrageenan-AuNPs after exposure to procaine reveal equilateral triangular-shaped particles with sides ranging narrowly from 19 to 27 nm preponderantly organized into J-type aggregates and unevenly distributed on the surface, as shown in Figure 8.

H-type aggregates are also confirmed by a deflection scan. Large voids are also present, with depths around 14.4 nm and 61.25 nm.

### 2.4. Potentiometric Detection of Procaine

The potentiometric response to procaine of the three sensors having the membrane composition presented in Table S2 is shown in Figures S1–S3.

As highlighted in Figures S1–S3, all the sensors have a potentiometric response to procaine in different areas of concentration.

**Figure 9:**
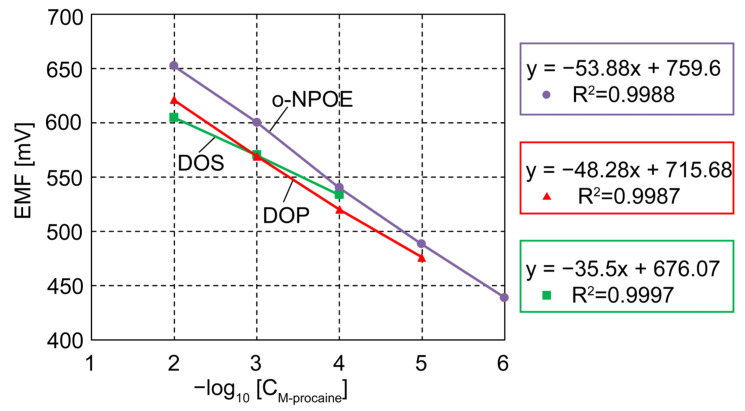
The linear range and slope for the three designed sensors plasticized differently with o-NPOE (purple line), DOS (green line), and DOP (red line).

The values of each sensor slope and the linear range are comparatively presented in Figure 9.

The selectivity coefficients, calculated by the separate solution method [38] or 10^−3^ M of procaine and interfering solutions, are also presented comparatively in Table 2.

As shown in Figure 9 and Table 2, the sensors are all procaine-selective, with good values of the selectivity coefficients. However, the best potentiometric results were obtained for sensor 2, with the membrane plasticized with o-NPOE. The sensor has a potentiometric answer to procaine in the range 10^−2^–10^−6^ M, with a slope of 53.88 mV/decade, very good values of the selectivity coefficients, and a detection limit of 7 × 10^−7^ M.

**Figure 10:**
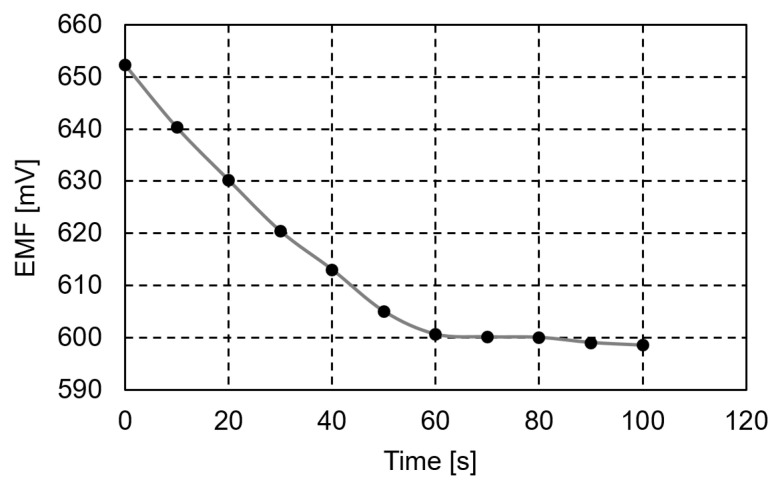
The response time of the membrane.

For the optimum composition of the sensor membrane, the response time of procaine solutions varying from 10^−4^ M to 10^−3^ M was 60 s, as presented in Figure 10.

**Figure 11:**
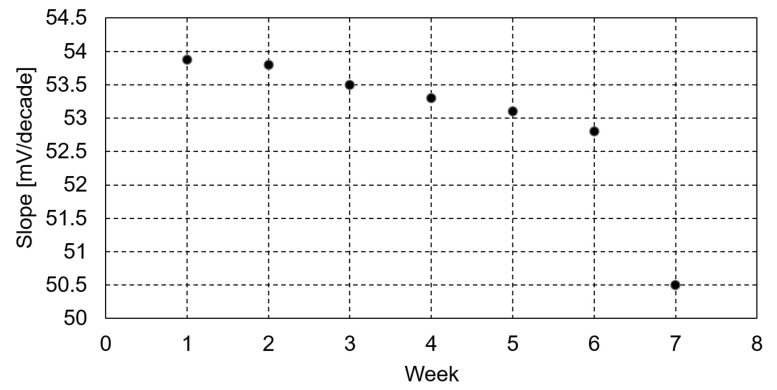
Stability in time (weeks) of the best-performing sensor.

The sensor was used for a period of 6 weeks without significant changes in the slope values, as depicted in Figure 11.

#### Proposed Mechanism for the Potentiometric Detection of Procaine

The magnitude of the dielectric constant of each plasticizer (o-NPOE, ε = 24; DOP, ε = 7; DOS, ε = 4) has important effects on membrane behavior. The higher polar o-NPOE plasticizer favors porphyrin aggregation in the membrane due to its –COOH hydrophilic function, and this behavior explains the well-fitted value of the Nernstian slope. The free active centers of the membrane are, in this way, more available for binding procaine by electrostatic interactions. This explanation is also supported by the AFM images (Figure 8) because triangular prismatic aggregation of porphyrins is usually found in more polar environments [39,40].

### 2.5. Analytical Applications

Sensor 2 was applied for the detection of procaine from procaine ampoules (pharmaceutically available) and analytically prepared laboratory synthetic samples, with very well-fitted results. The analytical test results are presented in Table 3.

## 3. Materials and Methods

The 5-(4-carboxyphenyl)-10,15,20-tris(4-methylphenyl)-porphyrin (5-COOH-3MPP) was previously synthesized and characterized [25]. Firstly, the 5-(4-methoxy-carbonyl-phenyl)-10,15,20-tris-(4-methyl-phenyl) porphyrin ester was prepared from methyl-4-formylbenzoate and 4-methylbenzaldehyde in a molar ratio of 1:3 by an improved Adler-Longo method. The ester was subsequently hydrolyzed in strong alkaline catalysis to furnish the carboxyl derivative.

### 3.1. Obtaining the 5-COOH-3MPP-k-Carrageenan-AuNPs Nanomaterial

The hybrid 5-COOH-3MPP-k-carrageenan-AuNPs material was obtained in the same manner as described in [25] by mixing together 5-(4-carboxyphenyl)-10,15,20-tris-(4-methyl-phenyl)-porphyrin and k-carrageenan in molar ratio 1:10 in DMF and then adding 36.2 mL of gold colloidal solution (c = 6.91 × 10^−4^ M) to 30 mL hybrid solution in order to obtain the final molar ratio porphyrin: k-carrageenan: AuNPs = 1:10:10. The gold nanoparticles of around 10–15 nm in diameter were obtained by reducing tetrachloroauric acid with citrate in hot water [41,42].

The purpose of the creation of this nanomaterial is the enlargement of the absorption domain and the amplification of the detection capabilities of the A_3_B porphyrin.

### 3.2. Polymeric Membrane Obtaining and Measurements

Three different procaine-selective membranes, with the composition presented in Table S2, have been prepared. The membrane composition includes porphyrin (5-COOH-3MPP), poly(vinyl)chloride (PVC) of high molecular weight, and either dioctylphtalate (DOP), bis(2-ethylhexyl), sebacate (DOS), or o-nitrophenyloctylether (o-NPOE) as plasticizers having different dielectric constants. Sodium tetraphenylborate (NaTPB) was used in each membrane as an additive (20% mol. relative to the ionophore). Tetrahydrofurane (THF) was used as a solvent for the membrane components. All the reagents were analytically pure.

The ingredients were mixed together and stirred for about 15–20 min to be dissolved. The obtained solutions were transferred to glass plates for the complete evaporation of THF at room temperature until flexible membranes with a thickness of around 15 µm were obtained. Afterward, round pieces of membranes 8 mm in diameter were cut and fixed to Fluka electrode bodies.

The potentiometric measurements were conducted using a HI 223 pH/mV at room temperature, and the cell composition is:

Hg|Hg_2_Cl_2_|bridge electrolyte|sample|ion-selective membrane|0.01 M KCl|AgCl, Ag.

All the sensors were conditioned for 24 h in a 10^−3^ M procaine hydrochloride solution. The procaine solutions, from 10^−2^–10^−7^ M, were prepared by weighing the proper amount and dissolving it in 2-(N-morpholino)ethanesulfonic acid (MES) buffer of pH = 5.5 (to establish the suitable conditions of water swallowing by the membrane) [43].

All the interfering solutions, 10^−3^ M, were prepared in the same way. The selectivity coefficients were calculated by a separate solution method using the theoretical value of the slope of 59.2 mV/decade. The detection limit of the sensor was determined at the intersection of the extrapolated linear range with the lowest concentration level segment of the calibration plot.

For the analytical applications, synthetic samples and pharmaceutical products were prepared as described before.

### 3.3. Apparatus

The recording of UV-vis spectra in 1 cm wide quartz cuvettes was performed on a V-650-JASCO spectrometer (Pfungstadt, Germany). A pH meter, the HI 98,100 Checker Plus from Hanna Instruments (Woonsocket, RI, USA), provided the pH values. Atomic force microscopy (AFM) images were obtained by deposition of samples on pure silica plates and visualization on a Nanosurf^®^ EasyScan 2 Advanced Research AFM microscope (Liestal, Switzerland) equipped with a piezoelectric ceramic cantilever. All FT-IR spectra were registered from KBr pellets in the range 4000–400 cm^−1^ on a JASCO 430 FT-IR (Hachioji, Tokyo, Japan) spectrometer.

## 4. Conclusions

The same A_3_B porphyrin structure was sensitive in both optical and potentiometric detection, changing the nature of the polymer in which it was incorporated. A hybrid nanomaterial containing 5-COOH-3MPP, k-carrageenan, and AuNPs was capable of optically detecting procaine in the concentration range from 5.76 × 10^−6^ M to 2.75 × 10^−7^ M, with a limit of detection (LOD) of 1.33 × 10^−7^ M, due to a chemical interaction between the donating amino groups from procaine and the accepting carboxyl group from porphyrin. The ion-selective electrode consisting of a PVC membrane plasticized with o-NPOE and the A3B porphyrin gave a complementary wider detected concentration domain in comparison with the optical method, from 10^−2^–10^−6^ M, a LOD of 7 × 10^−7^ M, a Nernstian slope of 53.88 mV/decade, a response time of 60 s, and stability over six weeks. The higher polar o-NPOE plasticizer favors porphyrin aggregation in the membrane, thus increasing the accessibility of procaine to the free active centers and its binding by electrostatic interactions. Covering the medical and biological domains of interest [5], both optical and potentiometric methods are highly appropriate for the investigation of procaine’s remaining toxicity. The methods prove to be accurate, simple, cost-effective, fast, and easy to apply, as they do not require expensive equipment. The potentiometric detection method provides a good response toward a wider procaine concentration range, whereas optical detection allows for the quantification of procaine in traces.

## Figures and Tables

**Table 1:** The relevant methods, materials, full concentration range, and limits of detection (LODs) in procaine quantification that have been reported in the last 5 years.

Detection Method	Materials	Fully Linear Detected Concentration Range [M]	LOD [M]	Ref.
Potentiometric detection	diamond/graphite hybrid with a molecular imprint membrane	4 × 10^−8^–2.5 × 10^−5^	1.5 × 10^−8^	[6]
Potentiometric titration	10^−1^ mol/L cerium(IV) sulfate solution in sulfuric acid	10^−2^–10^−4^	-	[7]
Potentiometric detection	5-COOH-3MPP in PVC membrane	10^−6^–10^−2^	7 × 10^−7^	This work
Cyclic voltammetry	electrode of carbon paste modified with multi-walled carbon nanotubes	2.4 × 10^−6^–10^−4^	62.0 × 10^−9^	[8]
UV-vis spectroscopy	Co(II)-tetra(3-hydroxyphenyl)porphyrin/AgNPs	5.39 × 10^−5^–28.04 × 10^−5^	1.1 × 10^−5^	[9]
UV-vis spectroscopy	5-COOH-3MPP + k-carrageenan + AuNPs nanomaterial	5.7 × 10^−6^–2.7 × 10^−7^	1.3 × 10^−7^	This work
Colorimetric detection	reaction with cerium (IV) sulfate tetrahydrate (sodium dodecyl sulfate as a sensitizer)	4.2 × 10^−6^–6.3 × 10^−4^	3.1× 10^−6^	[10]
Surface-enhanced Raman scattering (SERS) spectroscopic technique	gold nanoparticles	10^−3^–10^−8^	10^−10^	[11]
Surface-enhanced Raman scattering (SERS) spectroscopic technique	FTO electrodes modified with silver-decorated carbon nanospheres	10^−6^–10^−12^	10^−13^	[12]
SERS and EC-SERS	Pd-loaded highly reduced graphene oxide nanocomposite substrate	10^−2^–10^−7^	10^−8^	[13]
Magnetic solid-phase extraction coupled with high-performance liquid chromatography	-	8.46 × 10^−8^–2.12 × 10^−5^	1.68 × 10^−8^	[14]

**Table 2:** Selectivity coefficients of the sensors.

Sensor	Interferent (X)	Glucose	NH_4_Cl	KCl	Lactose	Urea	Procaine
1	logK_Procaine, X_	−2.52	−0.60	−0.63	−0.76	−0.76	0
2		−2.48	−2.36	−2.53	−2.50	−2.42	0
3		−0.58	0.62	−0.61	−0.65	−0.58	0

**Table 3:** Analytical applications of the procaine-selective sensor.

Samples	Potentiometric Detection (mg ± S ^a^)	Amount (mg)
Procaine ampoules	98 ± 1	100
Synthetic samples	197 ± 1.4	200

^a^ An average of determinations on three samples of the same origin.

## Data Availability

The data presented in this study are available on request from the first or corresponding author.

## Supplementary Materials

The following supporting information can be downloaded at https://www.mdpi.com/article/10.3390/ijms242417265/s1. References [40,44,45,46,47,48,49] are cited in Supplementary Materials.

## References

[1] Ayuse T., Kurata S., Ayuse T. (2020). Successful Dental Treatments Using Procaine Hydrochloride in a Patient Afraid of Local Anesthesia but Consenting for Allergic Testing with Lidocaine: A Case Report. Local Reg. Anesth..

[2] Coutant M., Malnkvist J., Keiser M., Foldager L., Herskin M.S. (2022). Piglets’ acute responses to procaine-based local anesthetic injection and surgical castration: Effects of two volumes of aesthetic. Front. Pain Res..

[3] Gradinaru D., Ungurianu A., Margina D., Moreno-Villanueva M., Bürkle A. (2021). Procaine-The Controversial Geroprotector Candidate: New Insights Regarding Its Molecular and Cellular Effects. Oxidat. Med. Cell. Longev..

[4] Ungurianu A., Margina D., Borsa C., Ionescu C., von Scheven G., Oziol L., Faure P., Artur Y., Burkle A., Gradinaru D. (2020). The radioprotective effect of procaine and procaine-derived product gerovital H3 in lymphocytes from young and aged individuals. Oxidat. Med. Cell. Longev..

[5] Malamed S.F., Malamed S.F. (2017). Section V: Intravenous Sedation Chapter 25-Pharmacology. Sedation.

[6] Zhu Y., Xu Y., Liu G. (2021). Electrochemical Detection of the Anesthetic Drug Procaine Hydrochloride Based on Molecularly Imprinted Polymer/Diamond-Graphite Composite Electrode. Int. J. Electrochem. Sci..

[7] Vladescu L., Moja D., Badea I.A., Ciomaga C. (2022). Determination of procaine hydrochloride by potentiometric titration. Rev. Roum. Chim..

[8] Haghighian F., Ghoreishi S.M., Attaran A., Kashani F.Z., Khoobi A. (2023). Electrochemical study for simultaneous detection of procaine hydrochloride and its metabolite in biological samples using a nanostructured strong sensor. Korean J. Chem. Eng..

[9] Lascu A., Palade A., Birdeanu M., Fagadar-Cosma E. (2019). Procaine detection using hybrids of cobalt-metalloporphyrin with gold and silver nanoparticles. J. Chem. Soc. Pak..

[10] Tantirungrotechai Y., Syananondh A., Youngvises N. (2023). Flow Analysis with Cerium (IV) Colorimetric Reagent for Determination of Procaine and Elucidation of the Color Species by Computational Investigation. Sci. Technol. Asia.

[11] Al-Saadi A.A., Haroon M., Popoola S.A., Saleh T.A. (2020). Sensitive SERS detection and characterization of procaine in aqueous media by reduced gold nanoparticles. Sens. Actuators B Chem..

[12] Haroon M., Abdulazeez I., Saleh T.A., Al-Saadi A.A. (2021). Electrochemically modulated SERS detection of procaine using FTO electrodes modified with silver-decorated carbon nanosphere. Electrochim. Acta.

[13] Haroon M., Ashraf M., Ullah N., Tahir M.N., Al-Saadi A.A. (2022). SERS and EC-SERS detection of local anesthetic procaine using Pd loaded highly reduced graphene oxide nanocomposite substrate. Spectrochim. Acta A Mol. Biomol. Spectrosc..

[14] Liang S.Y., Shi F., Zhao Y.G., Wang H.W. (2022). Determination of Local Anesthetic Drugs in Human Plasma Using Magnetic Solid-Phase Extraction Coupled with High-Performance Liquid Chromatography. Molecules.

[15] Han Q., Wang C., Liu P., Zhang G., Song L., Fu Y. (2021). Achieving synergistically enhanced dual-mode electrochemiluminescent and electrochemical drug sensors via a multi-effect porphyrin-based metal-organic framework. Sens. Actuators B Chem..

[16] Zhao B., Li Y., Zhao Y., Ma Y., Li F., Han H., Wang N., Wang X. (2022). A sensing platform based on zinc-porphyrin derinative in hexadecyl trimethyl ammonium bromide (CTAB) microemulsion for highly sensitive detection of theophylline. Spectrochim. Acta—A Mol. Biomol. Spectrosc..

[17] Lazoviskiy D.A., Mamardashvili G.M., Khodov I.A., Mamardashvili N.Z. (2020). Water soluble porphyrin-fluorescein triads: Design, DFT calculation and pH-change-triggered fluorescence response. J. Photochem. Photobiol. A Chem..

[18] Malecka K., Kaur B., Cristaldi D.A., Chay C.S., Mames I., Radecka H., Radecki J., Stulz E. (2021). Silver or gold? A comparison of nanoparticle modified electrochemical genosensors based on cobalt porphyrin-DNA. Bioelectrochemistry.

[19] Abouzar M.H., Poghossian A., Cherstvy A.G., Pedraza A.M., Ingebrandt S., Schöning M.J. (2012). Label-free electrical detection of DNA by means of field-effect nanoplate capacitors: Experiments and modeling. Phys. Status Solidi A.

[20] Comuzzi C., Marino M., Poletti D., Boaro M., Strazzolini P. (2022). New antimicrobial PVC composites. Porphyrins self-aggregation in tuning surface morphologies and photodynamic inactivation towards sustainable water disinfection. J. Photochem. Photobiol..

[21] Ghafuri H., Hanifehnejad P., Felfelian Z. (2023). Synthesis and characterization of porphyrin-modified chitosan biopolymer and its application in the degradation of methylene blue under visible light. J. Appl. Chem..

[22] Sewid F.A., Annas K.I., Dubavik A., Veniaminov A.V., Maslov V.G., Orlov A.O. (2022). Chitosan Nanocomposites with CdSe/ZnS Quantum Dots and Porphyrin. RSC Adv..

[23] Monteiro C.J.P., Neves M.G.P.M.S., Nativi C., Almeida A., Faustino M.A.F. (2023). Porphyrin Photosensitizers Grafted in Cellulose Supports: A Review. Int. J. Mol. Sci..

[24] Hino S., Funada R., Sugikawa K., Kawasaki R., Koumoto K., Suzuki T., Nagasaki T., Ikeda A. (2020). Mechanism Toward Turn-on of Polysaccharide–Porphyrin Complexes for Fluorescence Probes and Photosensitizers in Photodynamic Therapy in Living Cells. ChemMedChem.

[25] Epuran C., Fratilescu I., Macsim A.M., Lascu A., Ianasi C., Birdeanu M., Fagadar-Cosma E. (2022). Excellent Cooperation between Carboxyl-Substituted Porphyrins, k-Carrageenan and AuNPs for Extended Application in CO_2_ Capture and Manganese Ion Detection. Chemosensors.

[26] Birdeanu M., Fratilescu I., Epuran C., Murariu A.C., Socol G., Fagadar-Cosma E. (2022). Efficient Decrease in Corrosion of Steel in 0.1 M HCl Medium Realized by a Coating with Thin Layers of MnTa_2_O_6_ and Porphyrins Using Suitable Laser- Type Approaches. Nanomaterials.

[27] Mei Q., Shi Y., Hua Q., Tong B. (2015). Phosphorescent chemosensor for Hg^2+^ based on iridium(III) complex coordinated with 4-phenylquinazoline and sodium carbazole dithiocarbamate. RSC Adv..

[28] Borkhodoev V.Y. (2014). About the limit of detection in X-ray fluorescence analysis. J. Anal. Chem..

[29] Fulias A., Ledeti I., Vlase G., Popoiu C., Heghes A., Bilanin M., Vlase T., Gheorgheosu D., Craina M., Ferechide D. (2013). Thermal behaviour of procaine and benzocaine Part II: Compatibility study with some pharmaceutical excipients used in solid dosage forms. Chem. Cent. J..

[30] Mahmood W.A.K., Khan M.M.R., Yee T.C. (2014). Effects of reaction temperature on the synthesis and thermal properties of carrageenan ester. J. Phys. Sci..

[31] Lai W., Lau M.K., Chong V., Wong W.T., Leung W.H., Yu N.T. (2001). The metal–carbon stretching frequencies in methyl complexes of Rh, Ir, Ga and In with porphyrins and a tetradentate pyridine–amide ligand. J. Organomet. Chem..

[32] Hong T., Yin J.Y., Nie S.P., Xie M.Y. (2021). Applications of infrared spectroscopy in polysaccharide structural analysis: Progress, challenge and perspective. Food Chem. X.

[33] Lewis P.D., Lewis K.E., Ghosal R., Bayliss S., Lloyd A.J., Wills J., Godfrey R., Kloer P., Mur L.A. (2010). Evaluation of FTIR spectroscopy as a diagnostic tool for lung cancer using sputum. BMC Cancer.

[34] Hermán V., Takacs H., Duclairoir F., Renault O., Tortai J.H., Viala B. (2015). Core double–shell cobalt/graphene/polystyrene magnetic nanocomposites synthesized by in situ sonochemical polymerization. RSC Adv..

[35] D’Souza L., Devi P., Divya Shridhar M.P., Naik C.G. (2008). Use of Fourier Transform Infrared (FTIR) spectroscopy to study cadmium-induced changes in *Padina tetrastromatica* (Hauck). Anal. Chem. Insights.

[36] Sharma L., Kimura T. (2003). FT-IR Investigation into Miscible Interactions in New Materials for Optical Devices. Polym. Adv. Technol..

[37] Gloaguen E., Mons M., Schwing K., Gerhards M. (2020). Neutral Peptides in the Gas Phase: Conformation and Aggregation Issues. Chem. Rev..

[38] Umezawa Y., Buhlmann P., Umezawa K., Tohda K., Amemiya S. (2002). Potentiometric selectivity coefficients of ion-selective electrodes. Part I. Inorganic cations. Pure Appl. Chem..

[39] Fagadar-Cosma E., Lascu A., Shova S., Zaltariov M.-F., Birdeanu M., Croitor L., Balan A., Anghel D., Stamatin S. (2019). X-ray Structure Elucidation of a Pt-Metalloporphyrin and Its Application for Obtaining Sensitive AuNPs-Plasmonic Hybrids Capable of Detecting Triiodide Anions. Int. J. Mol. Sci..

[40] Fagadar-Cosma E., Tarabukina E., Zakharova N., Birdeanu M., Taranu B., Palade A., Creanga I., Lascu A., Fagadar-Cosma G. (2016). Hybrids formed between polyvinylpyrrolidone and an A_3_B porphyrin dye: Behaviour in aqueous solutions and chemical response to CO_2_ presence. Polym. Int..

[41] Muthukumar P., Abraham J.S. (2014). Gold nanoparticles decorated on cobalt porphyrin-modified glassy carbon electrode for the sensitive determination of nitrite ion. J. Colloid Interface Sci..

[42] Fringu I., Lascu A., Macsim A.M., Fratilescu I., Epuran C., Birdeanu M., Fagadar-Cosma E. (2022). Pt(II)-A2B2 metalloporphyrin-AuNPS hybrid material suitable for optical detection of 1-anthraquinonsulfonic acid. Chem. Pap..

[43] Vlascici D., Fagadar-Cosma G., Plesu N., Lascu A., Petric M., Crisan M., Belean A., Fagadar-Cosma E. (2018). Potentiometric sensors for iodide and bromide based on Pt(II)-porphyrin. Sensors.

[44] Hibbard A.J., Burnley H., Michaela J., Rubin H.N., Miera J.A., Reynolds M.M. (2020). Porphyrin-based metal-organic framework and polyvinylchloride composites for fluorescence sensing of divalent cadmium ions in water. Inorg. Chem. Commun..

[45] Itagaki Y., Deki K., Nakashima S.I., Sadaoka Y. (2005). Toxic gas detection using porphyrin dispersed polymer composites. Sens. Actuators B Chem..

[46] Özbek O., Isildak Ö. (2022). Potentiometric determination of copper(II) ions based on a porphyrin derivative. J. Chin. Chem. Soc..

[47] Amini M.K., Shahrokhian S., Tangestaninejad S. (1999). Porphyrins as carriers in poly(vinyl chloride)-based membrane potentiometric sensors for histamine. Analyst.

[48] Avossa J., Paolesse R., Di Natale C., Zampetti E., Bertoni G., De Cesare F., Scarascia-Mugnozza G., Macagnano A. (2019). Electrospinning of Polystyrene/Polyhydroxybutyrate Nanofibers Doped with Porphyrin and Graphene for Chemiresistor Gas Sensors. Nanomaterials.

[49] Özbek O. (2023). A potentiometric sensor for the determination of potassium in different baby follow–on milk, water, juice and pharmaceutical samples. J. Food Compos. Anal..

