# Supplementary material for: The Influence of the Nature of the Polymer Incorporating the Same A3B Multifunctional Porphyrin on the Optical or Electrical Capacity to Recognize Procaine
Source: Int J Mol Sci. 2023 Dec 8;24(24):17265. doi: 10.3390/ijms242417265 (PMC10743720; doi:10.3390/ijms242417265)
Supplement: Supplementary file 1 [file ijms-24-17265-s001.zip › ijms-2747910-supplementary.pdf]

The influence of the nature of the polymer incorporating the same A_3B multifunctional porphyrin on the optical or electrical capacity to recognize procaine

Anca Lascu [1], Dana Vlascici [2], Mihaela Birdeanu [3], Camelia Epuran [1], Ion Fratilescu [1] and Eugenia Fagadar-Cosma [1,*]

[1] Institute of Chemistry "Coriolan Dragulescu", Mihai Viteazu Ave. 24, 300223 Timisoara, Romania
[2] Faculty of Chemistry, Biology, Geography, West University of Timisoara, 4 Vasile Parvan Ave, 300223 Timisoara, Romania
[3] National Institute for Research and Development in Electrochemistry and Condensed Matter, Plautius Andronescu Street 1, 300224 Timisoara, Romania
* Correspondence: efagadar@yahoo.com or efagadarcosma@acad-icht.tm.edu.ro

Table S1. Polymers incorporating porphyrins as sensing materials for the detection of biologically active compounds.

Polymer and Incorporated porphyrin	Detected analyte	Concentration domain [M]	Advantages	Ref.
**Spectroscopic detection**				
Polyvinylpyrrolidone-5-(4-pyridyl)-10,15,20-tris(4-phenoxyphenyl)porphyrin	CO_2	$4.5 \times 10^{-5} - 1.5 \times 10^{-4}$	Can be used also as colorimetric sensor	[40]
Polyvinylchloride-$ZrOCl_2{\cdot}8H_2O$ -tetrakis(4-carboxyphenyl)porphine	Cd^{2+}	$0.01 - 0.1 \times 10^{-12}$	Water-stable MOF	[44]
Ethylcellulose with tetraphenylporphyrin; 5,10,15,20-tetrakis-(3,5-methoxyphenyl)porphyrin; 5,10,15,20-tetrakis(4-hydroxyphenyl)porphyrin; 5,10,15,20-tetrakis(4-carboxyphenyl)porphyrin	HCl, NO_2, SO_2	1.6 ppm; 0.5 ppm; 0.5 ppm	Sensitivity affected by humidity	[45]
**Electrochemical detection**				
Polyvinylchloride – 5,10,15,20-tetrakis(4-methoxycarbonylphenyl)porphyrin	Cu^{2+}	$1.0 \times 10^{-5} - 1.0 \times 10^{-1}$	- determination of copper in beverages	[46]
Polyvinylchloride – Iron(III) and manganese(III) tetraphenylporphyrins	histamine	$1 \times 10^{-6} - 1 \times 10^{-1}$	-High selectivity<br>-Wide pH range	[47]
Polystyrene and polyhydroxibutyrate, mesoporous graphitized carbon - tetraphenylporphyrin	Toluene Acetic acid	-	Capable to work at temperatures between 50 and 70 °C	[48]

Polyvinylchloride – 5,10,15,20–tetrakis(p–bromophenyl)porphyrin	K^+	$1 \times 10^{-5} – 1 \times 10^{-1}$	-Low cost<br>-High stability<br>-wide pH range	[49]

Table S2. Membrane composition of the potentiometric sensors (%).

Sensor	Electroactive material (5-COOH-3MPP)	PVC	Plasticizer	
1	1	33	DOS	66
2	1	33	o-NPOE	66
3	1	33	DOP	66

The potentiometric response of the obtained sensors

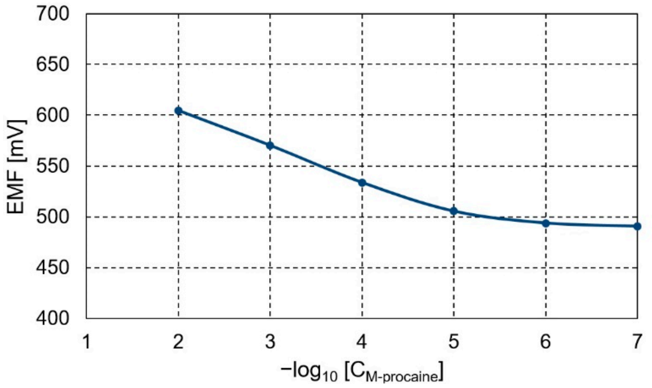

Figure S1. Potentiometric response of sensor 1 to procaine.

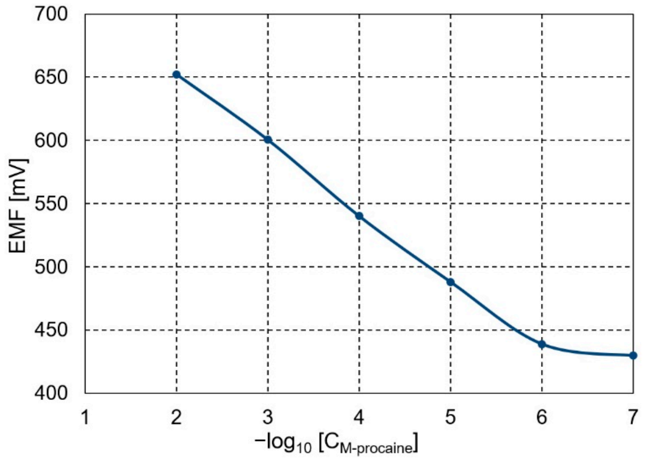

Figure S2. Potentiometric response of sensor 2 to procaine.

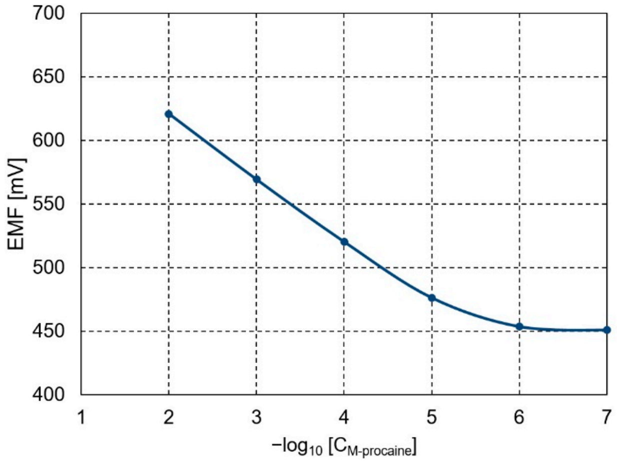

Figure S3. Potentiometric answer of sensor 3 to procaine.

References

40. Fagadar-Cosma, E.; Tarabukina, E.; Zakharova, Natalia; Birdeanu, M.; Taranu, B.; Palade, A.; Creanga, I.; Lascu, A.; Fagadar-Cosma, G. Hybrids formed between polyvinylpyrrolidone and an A_3B porphyrin dye: behaviour in aqueous solutions and chemical response to CO_2 presence. *Polym. Int*. **2016**, *65*, 200–209. https://doi.org/10.1002/pi.5047.

44. Hibbard, A. J.; Burnley, H.; Michaela, J.; Rubin, H.N.; Miera, J.A.; Reynolds, M. M. Porphyrin-based metal-organic framework and polyvinylchloride composites for fluorescence sensing of divalent cadmium ions in water. *Inorg. Chem. Commun.* **2020**, *115*, 107861. https://doi.org/10.1016/j.inoche.2020.107861

45. Itagaki, Y.; Deki, K.; Nakashima, S.I.; Sadaoka, Y. Toxic gas detection using porphyrin dispersed polymer composites. *Sens. Actuators B Chem*. **2005**, *108*, 393–397. https://doi.org/10.1016/j.snb.2004.10.055.

46. Özbek, O.; Isildak, Ö. Potentiometric determination of copper(II) ions based on a porphyrin derivative. *J. Chinese Chem. Soc.* **2022**, *69*, 1060-1069. https://doi.org/10.1002/jccs.202200168

47. Amini, M.K.; Shahrokhian, S.; Tangestaninejad, S. Porphyrins as carriers in poly(vinyl chloride)-based membrane potentiometric sensors for histamine. *Analyst*, **1999**, *124*, 1319–1322. https://doi.org/10.1039/A903500A.

48. Avossa, J.; Paolesse, R.; Di Natale, C.; Zampetti, E.; Bertoni, G.; De Cesare, F.; Scarascia-Mugnozza, G.; Macagnano, A. Electrospinning of Polystyrene/Polyhydroxybutyrate Nanofibers Doped with Porphyrin and Graphene for Chemiresistor Gas Sensors. *Nanomaterials* **2019**, *9*, 280. https://doi.org/10.3390/nano9020280.

49. Özbek, O. A potentiometric sensor for the determination of potassium in different baby follow–on milk, water, juice and pharmaceutical samples. *J. Food Compos. Anal*. **2023**, *115*, 104937. https://doi.org/10.1016/j.jfca.2022.104937
